# Platelet-Rich Plasma-Derived Exosome Olfactory Cleft Injection for Treating Traumatic Olfactory Dysfunction

**DOI:** 10.3390/diagnostics16101547

**Published:** 2026-05-19

**Authors:** Rong-San Jiang

**Affiliations:** 1 Department of Otolaryngology, Tungs’ Taichung MetroHarbor Hospital, Taichung 435403, Taiwan; rsjtaiwan@yahoo.com.tw; 2 Department of Medical Research, Taichung Veterans General Hospital, Taichung 407219, Taiwan; 3 School of Medicine, Chung Shan Medical University, Taichung 402367, Taiwan

**Keywords:** head trauma, olfactory cleft, olfactory dysfunction, platelet-rich exosome, platelet-rich plasma

## Abstract

**Background:** Recently, platelet-rich plasma (PRP) nasal injections have been used to treat traumatic olfactory dysfunction related to Coronavirus disease 2019. Currently, however, platelet-rich plasma-derived exosomes (PREs) are emerging as key effectors of PRP activity and, when compared with PRP, exhibit superior stability and minimal immunogenicity. **Case Presentation:** We described a case of a 27-year-old woman who lost her olfactory function after hitting her head on 8 July 2022. A smell test demonstrated complete loss of olfactory function. She performed olfactory training and received PRP olfactory cleft injections three times, allowing her olfactory function to gain mild improvement. Subsequently, she received four additional PRE olfactory cleft injections, allowing her olfactory function to improve even further. **Conclusions:** This study presents a case of traumatic olfactory dysfunction treated with PRE olfactory cleft injections, but no real conclusions can be drawn regarding the efficacy of PRE olfactory cleft injection on traumatic olfactory dysfunction by a single case report.

## 1. Introduction

Head trauma is one of the leading causes of persistent olfactory dysfunction, and its pathophysiology is often multifactorial [1]. The pathophysiology of traumatic olfactory dysfunction includes the fact that the olfactory nerves are sheared on the cribriform plate and the central olfactory structures are injured [2]. The prevalence of traumatic olfactory dysfunction varies according to the severity of the injury, ranging from approximately 5–30% after mild traumatic brain injury to more than 50% in patients with severe head trauma [3,4]. Although approximately one third of patients may recover spontaneously, recovery is often incomplete and usually occurs within the first year after trauma; therefore, persistent anosmia beyond 1 to 2 years generally indicates a poorer prognosis [5,6].

A standard treatment modality for traumatic olfactory dysfunction has not yet been established [7]. Several treatments, including steroids and zinc, have been used to treat traumatic olfactory dysfunction; however, their effects are still controversial [6,8]. Olfactory training (OT) has been used to treat various types of olfactory loss [9,10,11]. However, its effect on traumatic olfactory dysfunction has been reported to be unsatisfactory [12,13,14].

Since the Coronavirus disease 2019 (COVID-19) pandemic, there has been a considerable increase in the prevalence of olfactory dysfunction [15,16]. Platelet-rich plasma (PRP) nasal injections have been used to treat olfactory loss caused by COVID-19 infection [17,18]. PRP contains growth factors such as transforming growth factor beta (TGF-beta), epidermal growth factor (EGF), vascular endothelial growth factor (VEGF), nerve growth factor (NGF), and insulin-like growth factor (IGF) and has been considered to have pro-regenerative properties [19,20]. A studythat included fourrandomized controlled trials concluded that PRP olfactory cleft injection was safe and offered promising results for COVID-19-related olfactory dysfunction [21]. PRP olfactory cleft injection was also used to treat traumatic olfactory dysfunction in two small cohort studies that reported safe and promising results [22,23]. Recently, we conducted a prospective randomized clinical trial using PRA and hyaluronic acid (HA) injection fluid to clarify its effect on traumatic olfactory dysfunction [24]. HA is a high molecular weight glycosaminoglycan that serves as the backbone of proteoglycans in the extracellular matrix [25]. Acid may possibly induce the release of growth factors [26,27]. PRP and HA combination therapy has been reported to have the potential to produce superior results compared with PRP therapy alone [25].

Active platelet activation has been reported to induce the secretion of extracellular vesicles, including exosomes. Platelet-rich plasma-derived exosomes (PRE) have been shown to be helpful in both tissue repair and regeneration, and PREs also have anti-inflammatory function and can regulate cellular bioactivity [28]. PREs can transport those important factors, such as the transforming growth factor beta 1, platelet-derived growth factor BB, vascular endothelial growth factor, and stromal cell-derived factor 1 [29]. Based on the regenerative properties of PREs, the aim of this study was to present one case of traumatic olfactory dysfunction that was treated with PRE olfactory cleft injection. This study was approved by the Institutional Review Board(III) of Taichung Veterans General Hospital (Protocol code:CE26149C, date of approval: 15 April 2026), and written informed consent was obtained from the patient.

## 2. Case Report

**Figure 1:**
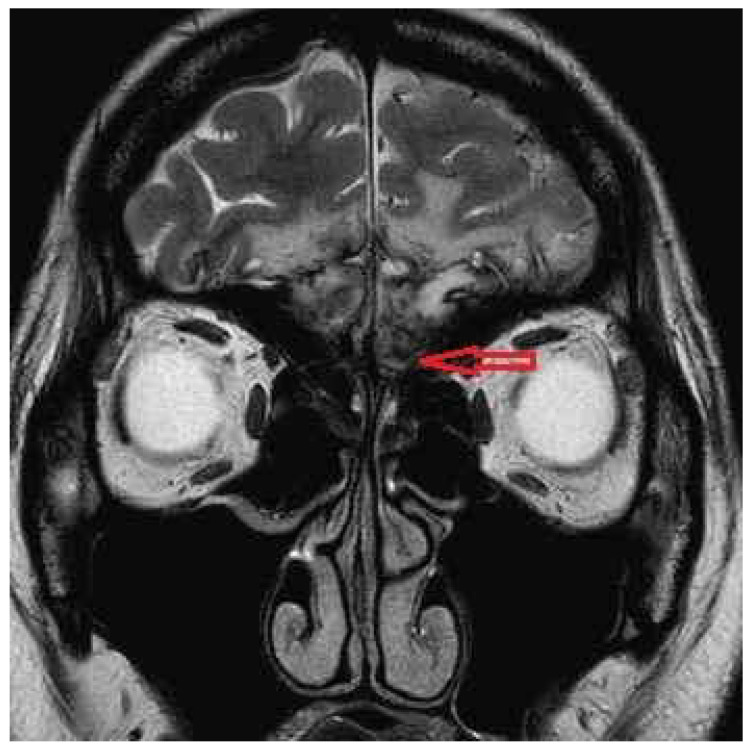
Magnetic resonance imaging showed injury to the base of the frontal lobe (red arrow).

A 27-year-old female patient had lost her olfactory function after she hit her head on 8 July 2022. A10-point Visual Analog Scale (VAS) and the phenyl ethyl alcohol (PEA) odor detection threshold test were used to assess her smell function [30]. On 24 August 2022, the test results showed that VAS score was 1, and the bilateral and unilateral PEA thresholds were each −1. Her brain magnetic resonance imaging (MRI) revealed that her bilateral olfactory bulbs were injured (Figure 1). She was treated with zinc gluconate (10mg, three times a day) along with OT, which used 4 separate bottles of PEA, lemon, eucalyptus, and clove oil. Patients were told to sniff each odorant for 10 s, twice a day. Until 5 September 2023, her olfactory function did not improve. The test results showed that the VAS score was 2, and the bilateral and unilateral PEA thresholds were each −1.

Subsequently, she underwent three PRA and HA olfactory cleft injections as additional treatment during the period from September 2023 to October 2024. Subsequently, she felt that her olfactory function had improved slightly (VAS: 4), but the PEA test performed on 27 November 2024 revealed that her bilateral and unilateral PEA thresholds were still at −1.

**Figure 2:**
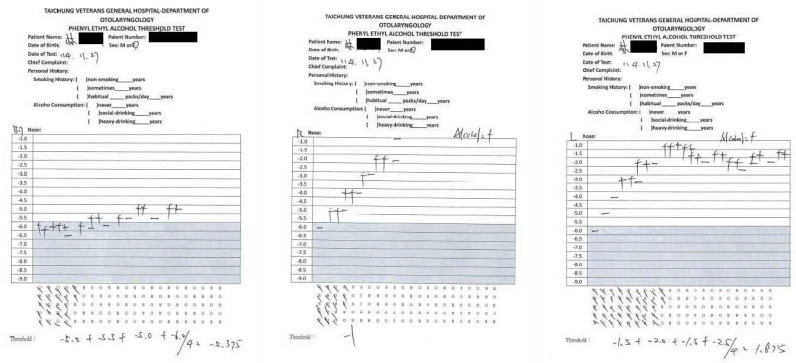
Phenylethyl alcohol (PEA) odor detection threshold test claimed that the bilateral PEA threshold was −5.375, the right unilateral PEA threshold was −1, and the left unilateral PEA threshold was −1.875.

**Figure 3:**
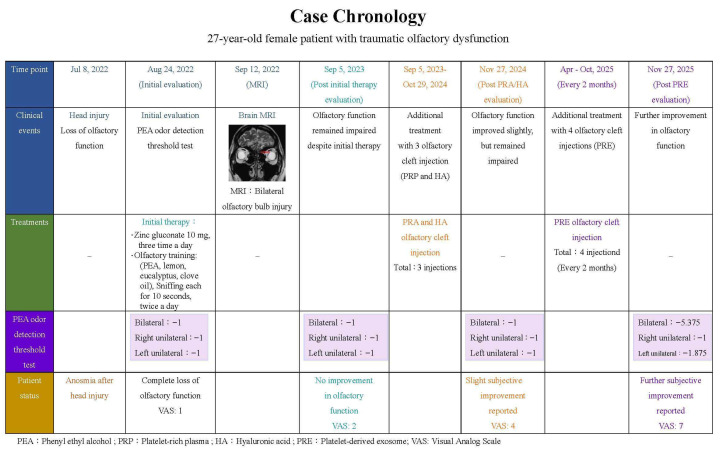
Chronology of smell tests and treatments.

To improve her olfactory function, she opted to receive four additional PRE olfactory cleft injections during the period from April to October 2025 at 2-month intervals. After all four PRE olfactory cleft injections had been completed, the patient felt that her olfactory function had improved further (VAS: 7). On 27 November 2025, her bilateral threshold improved to −5.375, her right unilateral threshold was −1, and her left unilateral threshold improved to −1.875 (Figure 2). Figure 3 shows the chronology of smell tests and treatments.

Since PRE olfactory cleft injections were performed under local anesthesia, the patient was asked about the severity of the pain of the procedures. Although she felt that the injection procedures were a little painful, she considered that the procedures were tolerable. After completing olfactory cleft injections, possible adverse effects were observed over a half-hour period. Mild epistaxis was observed after injection procedures, but no further procedure was performed to stop epistaxis. No headache was complained of. During each follow-up, she was asked about any adverse event related to PRE olfactory cleft injections, including a change in olfactory function, but no other severe adverse event was reported. She also received a rhinoscopic examination and PEA threshold test. No signs of wound infection, nasal crusting, or worsening of the smell were observed.

### 2.1. Tests of Olfactory Function

The olfactory function was evaluated using a 10-point VAS and the bilateral and unilateral PEA odor detection threshold test.

The patient reported her olfactory function using a 10-point VAS, where 0 indicated a complete loss of olfactory function and 10 represented normal olfactory function.

The PEA odor detection threshold test used in this study is also commercially available as the Snap & Sniff^®^ olfactory test system [30]. Different concentrations of a PEA odorant were prepared to measure the odor detection threshold of each patient. The strongest concentration was prepared with one cup of PEA solution mixed with 9 cups of mineral oil to create a concentration of PEA at 10^−1^ log vol/vol. Then, the concentration of PEA was diluted with mineral oil in half-log steps. The weakest concentration was at 10^−9^ log vol/vol.

A 2-alternative forced-choice single-staircase procedure was used to decide the PEA threshold. Testing procedures began with two sniff bottles containing either the PEA odorant at 10^−6^ log vol/vol or just mineral oil alone. The two bottles were opened in random order and placed under the patient’s nose. The patient then needed to indicate which of the two bottles had the strongest odor. Five successive correct answers had to be acquired before the PEA concentration was lowered to the next level; however, an incorrect answer shifted the PEA concentration to the next higher level. After the initial 5 successive correct answers had been acquired, only 2 successive correct identifications of PEA bottles were required during the following steps. The test was completed when 7 reversals of PEA concentrations had been reached, with the PEA threshold then estimated using the geometric mean of the last 4 reversed concentrations. The threshold scores ranged from −1 to −9.

The PEA odor detection threshold test was performed birhinally and unirhinally in this study. When the PEA threshold test was performed unirhinally, one nostril was blocked with a pad. In this study, the bilateral PEA test was first performed, and then the unilateral test was performed successfully using the right or left nostril in a random order.

### 2.2. Brain Magnetic Resonance Imaging

Brain MRI studies were performed using a 1.5 Tesla Excite MRI system (GEMS, Milwaukee, WI, USA) with a quadrature head coil. Routine imaging pulse sequences consisted of axial T1-weighted images and FLAIR images, along with axial and coronal T2-weighted fast spin-echo images. Contrast-enhanced T1-weighted images with axial and coronal sections were acquired. After a sagittal localization scan, 2 to 2.5 mm thick T2-weighted coronal and sagittal images (both TR = 5000 ms, TE = 106 ms, NEX = 2, Matrix = 256 × 256) without an interslice gap were obtained with a field of view of 12 cm.

### 2.3. Preparation of the PRA and HA Injection Fluid

The injection fluid of PRA and HA was prepared using a Cellular Matrix^®^ A-CP-HA Kit (Regen Lab SA, Le Mont-sur-Lausanne, Switzerland). A Cellular Matrix^®^ A-CP-HA tube was used as a centrifuge tube that contained 2 mL of natural, non-cross-linked HA, at a concentration of 20 mg/mL, in addition to a thixotropic cell separation gel and sodium citrate anticoagulant solution. Initially, 6 cc of whole blood was drawn from each patient and injected into a Cellular Matrix^®^ A-CP-HA tube. This tube was inverted 5 times to mix the whole blood with the sodium citrate anticoagulant solution. The tube was then centrifuged at 3430 rpm for 5 min. During centrifugation, the separator gel separated red blood cells from other blood components, with the HA migrating to the top of the gradient. Subsequently, the tube was inverted 20 more times to mix PRP and HA. Finally, 6 cc of the mixed fluid was drawn using an empty syringe for later injection.

### 2.4. PRA and HAInjection Procedures

The PRR and HA olfactory cleft injections were performed under an endoscope. Before injection, nasal cavities were packed with cotton pledgets soaked with a 2% xylocaine/epinephrine solution. After removing the pledgets, a 2% xylocaine/epinephrine solution was injected into the nasal septum and the middle turbinate. Then an elevator was used to lateralize the middle turbinate to expose the olfactory cleft. Finally, approximately 1–2 cc of the PRA and HA fluid was injected into the upper middle region of the right and left nasal septum, while approximately 1 cc of the fluid was injected into the medial surface of the middle turbinates of the right and left.

### 2.5. Preparation of the PRE Injection Fluid

**Figure 4:**
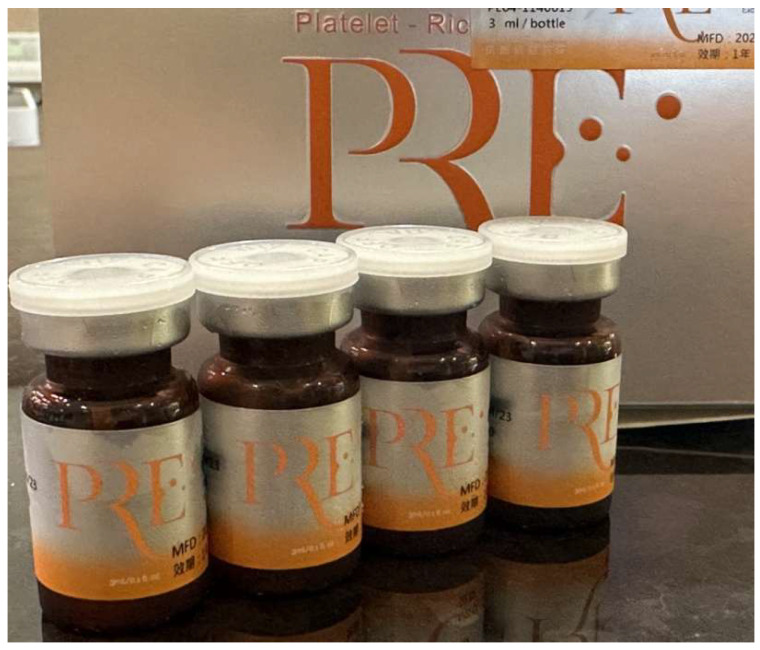
Platelet-rich exosomes were packed in 4 bottles.

For the preparation of the PRE injection fluid, 150 mL of peripheral blood was drawn from the patient and stored in blood bags. The bags were then sent to a local company (BIONET; Therapeutics Corp., Taipei, Taiwan) to manufacture exosomes. The procedures for both the collection and purification of exosomes have been outlined in a previous article [31]. Briefly, exosomes were manufactured using a one-stop production process that included bioreactors, continuous centrifugation, TFF concentration, and automatic filling systems. Qualitative and quantitative quality control covered size, purity, concentration, related proteins, and miRNA standardization. After quality inspection, the exosomes were packed into 4 bottles, each bottle containing 3 cc PRE fluid (Figure 4). The bottles were shipped to the clinic and stored in a refrigerator set at 4 degrees.

### 2.6. Injection Procedures

**Figure 5:**
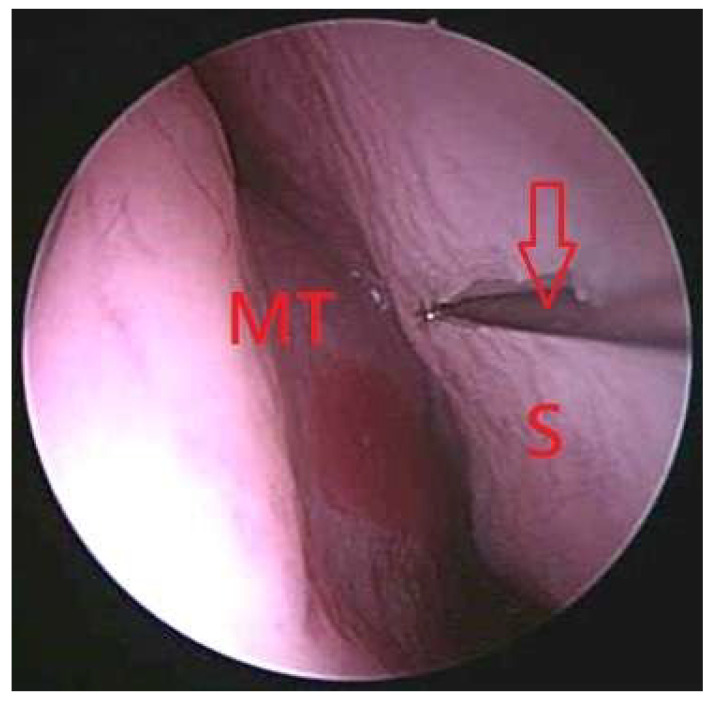
Platelet-rich exosome fluid was injected into the nasal septum. MT: middle turbinate; S: nasal septum; arrow: injection needle.

The PRE olfactory cleft injections were performed under an endoscope. Before injection, nasal cavities were packed with cotton pledgets soaked with a 2% xylocaine/epinephrine solution. After removing the pledgets, a 2% xylocaine/epinephrine solution was then injected into the nasal septum and the middle turbinate using a 23-gauge spinal needle. Subsequently, an elevator was used to lateralize the middle turbinate to expose the olfactory cleft. Finally, approximately 2 cc of PRE fluid was injected into the upper middle region of the right nasal septum submucosally using a 23-gauge spinal needle, while approximately 1 cc of the fluid was injected into the medial surface of the right middle turbinates submucosally using a 23-gauge spinal needle. The same procedure was performed in the left nasal cavity (Figure 5).

## 3. Discussion

Head trauma is a common etiology of sensorineural olfactory dysfunction, having a generally poor prognosis of traumatic olfactory dysfunction [6]. Up to now, there has been no standard treatment modality for traumatic olfactory dysfunction [7].

Recently, PRP olfactory cleft injection has been used to treat traumatic olfactory dysfunction, as revealed in several reports [22,23,24,32]. In our previous preliminary report, we treated 28 patients experiencing traumatic olfactory dysfunction by injecting a combination of PRP and HA into the olfactory cleft, and 85.7% of the patients reported subjective improvement in their olfactory function [22]. Lechien [23] reported on a preliminary study on the use of PRP for traumatic olfactory dysfunction. His patients were divided into 33 traumatic patients who had received PRP olfactory cleft injection and underwent OT for 3 months and 40 patients with olfactory dysfunction who only underwent OT for 3 months. At post-treatment follow-up, 66.7% of patients who had received PRP olfactory cleft injection reported subjective improvement in olfactory function compared with a 30.5% improvement rate in patients who only underwent OT. In our prospective randomized clinical trial [24], 37 traumatic patients received PRP and HA olfactory cleft injection and underwent OT for 3 months and 40 traumatic patients in the control group only underwent OT for 3 months. In total, 75.7% of the patients in the PRP and HA group reported subjective improvement in olfactory compared with only 50% of the patients in the control group, revealing that the improvement rate was significantly better in the PRP and HA group than in the control group. However, many patients are still unsatisfied with the results of their PRP injection treatments. Therefore, other treatment modalities are still necessary to treat these particular patients who are experiencing traumatic olfactory dysfunction.

Wang and Patel [32] reported on a male patient experiencing traumatic olfactory dysfunction who had received PRP injections into his bilateral olfactory clefts. His score on the University of Pennsylvania Smell Identification Test increased from 12 before injections of PRP to 19 one month after the third injection of PRP.

In this case, our patient had failed three times of PRP and HA olfactory cleft injection, but her PEA threshold improved from −1 to −5.375 after PRE olfactory cleft injection. Although the PEA threshold test is widely used for the smell test and is commercially available as the Snap & Sniff test, a minimally clinically important difference for PEA thresholds has not yet been universally defined [30]. Additionally, the reliability of the test and the re-test is not reported in the Snap & Sniff test administrative manual. Therefore, the clinical interpretation of changes in the PEA threshold must be cautious. Regarding the discrepancy between the results of unilateral and bilateral tests, the change in bilateral threshold may reflect enhanced central sensory integration or the summation effects of unilateral testing. On the contrary, unilateral performance can remain limited due to possible side-specific deficits after head trauma.

PREs have been reported to help tissue repair and regeneration, and they also have anti-inflammatory function and can regulate cellular bioactivity [28]. The mechanistic differences between PRP and PRE may include several aspects. Exosomes are nanoscale extracellular vesicles that can fuse directly with recipient cells and deliver molecular cargo intracellularly. Therefore, its biologic effect may be more targeted and durable than the extracellular diffusion of soluble PRP growth factors [33]. PREs contain selected proteins, lipids, messenger RNAs, and microRNAs rather than the full plasma environment. This selective enrichment may be important if previous PRP failed because growth factors degraded too quickly, inflammatory plasma proteins counteracted the benefit, tissue penetration was insufficient, or neuronal signaling pathways needed intracellular reprogramming rather than extracellular stimulation [34]. Because exosomes are extremely small, they can diffuse through mucus, extracellular matrix, and damaged neuroepithelium more effectively than bulk PRP preparations [35].

### Limitations

There are several limitations in this report. Only one case report did not demonstrate the effect of PRE olfactory cleft injection in the treatment of traumatic olfactory dysfunction. More cases or prospective randomized trials are needed to evaluate its efficacy. Furthermore, how many PRE olfactory cleft injections are needed to treat traumatic olfactory dysfunction has not yet been determined. Third, in this report, it cannot be evaluated whether an earlier injection had a better effect on the treatment of traumatic olfactory dysfunction. Fourth, our patient was only followed for a short interval after receiving PRE injections. Therefore, the long-term effect and safety of PRE olfactory cleft injection in the treatment of traumatic olfactory dysfunction still require further study. Finally, the efficacy of PRE is significantly influenced by their preparation methods, primarily because different techniques can lead to substantial variations in their composition and concentration [36]. The manufacturer did not provide detailed information onthePRE preparation methods used in this case. This makes it extremely challenging to compare the results of this case with others.

## 4. Conclusions

This study presents a case of traumatic olfactory dysfunction in which PRP olfactory cleft injections did not result in any satisfactory improvement in olfactory function. However, more patients with different injection modalities and a longer follow-up period are still required to better investigate the exact effects and safety of PRE olfactory cleft injection in the treatment of traumatic olfactory dysfunction. Moreover, because there are several limitations in our report, our results should be interpreted with caution. Therefore, no real conclusions can be made regarding the efficacy of PRE olfactory cleft injection on traumatic olfactory dysfunction.

## Data Availability

The data presented in this study are available on request from the corresponding author.
